# Metabolism of Genipin in Rat and Identification of Metabolites by Using Ultraperformance Liquid Chromatography/Quadrupole Time-of-Flight Tandem Mass Spectrometry

**DOI:** 10.1155/2013/957030

**Published:** 2013-03-19

**Authors:** Yue Ding, Jian-Wei Hou, Yong Zhang, Li-Ying Zhang, Tong Zhang, Yi Chen, Zhen-Zhen Cai, Li Yang

**Affiliations:** ^1^Experiment Center for Teaching and Learning, Shanghai University of Traditional Chinese Medicine, Shanghai 201203, China; ^2^School of Pharmacy, Shanghai University of Traditional Chinese Medicine, Shanghai 201203, China; ^3^Institute of Chinese Materia Medica, Shanghai University of Traditional Chinese Medicine, Shanghai 201203, China

## Abstract

The *in vivo* and *in vitro* metabolism of genipin was systematically investigated in the present study. Urine, plasma, feces, and bile were collected from rats after oral administration of genipin at a dose of 50 mg/kg body weight. A rapid and sensitive method using ultraperformance liquid chromatography coupled with electrospray ionization quadrupole time-of-flight tandem mass spectrometry (UPLC-Q/TOF MS) was developed for analysis of metabolic profile of genipin in rat biological samples (urine, plasma, feces, and bile). A total of ten metabolites were detected and identified by comparing their fragmentation patterns with that of genipin using MetaboLynx software tools. On the basis of the chromatographic peak area, the sulfated and glucuronidated conjugates of genipin were identified as major metabolites. And the existence of major metabolites G1 and G2 was confirmed by the *in vitro* enzymatic study further. Then, metabolite G1 was isolated from rat bile by semipreparative HPLC. Its structure was unambiguously identified as genipin-1-o-glucuronic acid by comparison of its UV, IR, ESI-MS, ^1^H-NMR, and ^13^C-NMR spectra with conference. In general, genipin was a very active compound that would transform immediately, and the parent form of genipin could not be observed in rats biological samples. The biotransformation pathways of genipin involved demethylated, ring-opened, cysteine-conjugated, hydroformylated, glucuronidated, and sulfated transformations.

## 1. Introduction

Genipin is an aglycone derived from an iridoid glycoside called geniposide, which is present in the fruit of *Gardenia jasminoides* Ellis. Intestinal bacteria in animals can transform geniposide to its aglycone genipin [1]. In our laboratory, geniposide could be transformed into genipin by immobilized *β*-Glucosidase in a two-phase aqueous-organic system [2]. Genipin has been proven to possess multiple bioactivities including antitumor [3, 4], neuroprotective [5], choleretic [6], and anti-inflammatory effects [7–9]. It is also an excellent natural cross-linker for proteins, collagen, gelatin, and chitosan cross-linking. It is much less toxic than glutaraldehyde and many other commonly used synthetic cross-linking regents [10]. Genipin-chitosan delivery systems are useful to control the release of such different drugs as clarithromycin, tramadol hydrochloride, and low-molecular-weight heparin loaded in spray-dried microspheres [11].

 In our previous studies, the parent form of genipin could not be detected directly in all plasma specimens of rats as it had been transformed to other forms such as conjugated genipin. The conjugated genipin could be hydrolyzed with sulfatase to genipin [12]. So, it can be assumed that some metabolites could exert stronger bioactivities. Therefore, investigation of the metabolites of genipin is of great significance in elucidation of its pharmacological mechanisms and discovering novel drugs from the metabolites. However, the metabolism of genipin has not being fully investigated as no report has been seen in the literature that comprehensively and comparatively investigated the *in vivo* and *in vitro* metabolic profile of this compound.

 In the present study, metabolism of genipin was investigated systematically by using ultraperformance liquid chromatography coupled with electrospray ionization quadrupole time-of-flight tandem mass spectrometry (UPLC-Q/TOF MS). The *in vivo* metabolites were detected in plasma, bile, urine, and feces. One phase I metabolite and nine phase II metabolites were detected totally and their structures were identified. The *in vitro* incubation and enzymatic hydrolysis analysis also confirmed the existence of two phase II metabolites. And one major phase II metabolite (genipin-1-o-glucuronic acid) had been prepared by preparative chromatography technology, and its structure was unambiguously identified by comparison of its UV, IR, ESI-MS, ^1^H-NMR, and ^13^C-NMR spectra with conference [13]. The biotransformation pathways of genipin involved demethylated, ring-opened, cysteine-conjugated, hydroformylated, glucuronidated, and sulfated transformations.

## 2. Experimental 

### 2.1. Chemicals and Reagents

 Genipin with a purity of 98.0% by HPLC was supplied by Wako Pure Chemical Industries Ltd (Japan). Sulfatase (type H-1 from Helix pomatia, containing 26.290 units/g) and *β*-glucuronidase (type B-1, from bovine liver, containing 1.240.000 units/g), UDPGA (Uridine 5′-diphosphoglucuronic acid trisodium salt), alamethicin (from Trichoderma viride), and saccharolactone were purchased from Sigma Chemical Co. Ltd (St Louis, MO, USA). HPLC-grade methanol and acetonitrile were purchased from Merck (Darmstadt, Germany). Ultrapure water was prepared with Milli-Q Ultrapure water purification system (Millipore, Bedford, MA, USA) and used for all analyses. Pooled human liver microsomes (HLM) protein (10 mg/mL) was supplied by the Research Institute for Liver diseases (Shanghai, CA). Potassium phosphate and MgCl_2_ (magnesium chloride) were supplied by Sinopharm Chemical Reagents Co. Ltd. 

### 2.2. *In Vivo* Experiment

#### 2.2.1. Animals

 Twenty-four male Sprague-Dawley rats weighing 180–220 g were obtained from the Laboratory Animal Center of the Shanghai University of Traditional Chinese Medicine (TCM). These rats were kept in an air-conditioned animal quarter at a temperature of 22–24°C and a relative humidity of 50 ± 10% and had access to the standard laboratory food and water. The rats were divided into four groups at random. Rats in the first group (*n* = 6) were kept in metabolic cages. Before the experiment, the blank urine and feces samples from each rat were collected for 12 h using a metabolic cage, and 300 *μ*L blood samples were collected from the suborbital veniplex in heparinized tubes. Then, rats were given a single dose of genipin solution at 50 mg/kg of body weight by gavage into the stomach. After administration, food and water were provided freely, and the drug-containing urine and feces samples were collected during the time of 0–24 h. The drug-containing blood samples were collected at 10, 60, 240, and 720 min after administration. Rats in the second group (*n* = 6) were orally administered distilled water at 2 mL/100 g. The blank bile samples were collected by bile duct cannulation surgery. Rats in the third group (*n* = 6) were orally administered genipin solution at 50 mg/kg of body weight, and the drug-containing bile samples were collected. Rats in the fourth group (*n* = 6) were given a single dose of genipin solution at 10 mg/kg by intravenous administration, and the blank blood samples were collected before administration, while the drug-containing blood samples were collected at 10, 60, 240, and 720 min after administration of genipin. Animal experiments were carried out in accordance with the local institutional guidelines for animal care of Shanghai University of Traditional Chinese Medicine. Plasma was separated from blood placed in heparinized Eppendorf tubes after centrifuging at 4000 ×g for 10 min. All samples were kept at −80°C.

#### 2.2.2. Sample Preparation

Plasma, urine, and bile 400 *μ*L samples were precipitated with 3 volumes of methanol. The supernatant was separated after vortex-mixed and centrifuging. Feces 500 mg 0–48 h sample was grounded and extracted by ultrasonication with 1 mL water for 30 min each time, and the extracted solution was precipitated with 3 volumes of methanol. The supernatant was separated after vortex-mixed and centrifuging. Extracting solutions with different pretreatment methods described the earlier were all dried under nitrogen gas over a water bath of 37°C. The residues were reconstituted in 100 *μ*L solution consisted of acetonitrile and water (15 : 85) and centrifuged at 10000 ×g for 10 min prior to analysis.

### 2.3. *In Vitro* Incubation Experiment

Glucuronidation catalyzed by the UDP-glucuronosyltransferases (UGTs) was a major pathway for drug metabolism and elimination in humans. We added 100 *μ*L of genipin (2.25 *μ*g/mL) dissolved in methanol to empty incubation tubes (1.5 mL polypropylene microcentrifuge tube) and dried it under nitrogen gas over a water bath of 37°C. We placed the incubation tubes on ice and added 50 *μ*g of pooled human liver microsomes (HLM) protein, 2.5 *μ*g alamethicin (2.5 *μ*g/*μ*L methanol; 50 *μ*g alamethicin/mg microsomes protein), and balanced to a volume of 50 *μ*L with 50 mM potassium phosphate buffer (pH 7.5, which included protein at final concentration of 0.5 mg/mL). We preincubated the tubes at 37°C for 5 min. We started reaction by adding 50 *μ*L of UDPGA cofactor solution (including 0.645 mg UDPGA, 10 *μ*L 50 mM magnesium chloride solution, 10 *μ*L 50 mM saccharolactone, 25 *μ*L 100 mM potassium phosphate buffer, pH 7.5., and 5 *μ*L water), mixed the tube by gentle flicking, capped tube, and incubated it for up to 6 h. The incubated solution was immediately treated with stop solution (ice-cold acetonitrile) after vortex and centrifuged at 14000 ×g for 10 min. We transferred 200 *μ*L supernatant to tubes, dried it under nitrogen gas over a water bath of 37°C, then reconstituted it with 100 *μ*L solution consisted of acetonitrile: water (15 : 85), centrifuged at 10000 ×g for 10 min prior to analysis. To better identify the glucuronide metabolite peak, there were also three negative controls that (1) contained no UDPGA, (2) contained no substrate, and (3) were not incubated. The samples were analyzed by using UPLC-Q/TOF MS.

### 2.4. Enzymatic Hydrolysis Analysis

 Enzymatic hydrolysis analysis was performed when searching and confirming the presence of phase II metabolites. The experiment initiated with collecting the drug-containing bile samples of rats in the third group. A 400 *μ*L bile sample was mixed with 50 *μ*L of *β*-glucuronidase or sulfatase (1000 units/mL in pH 5.0 acetate buffer) and incubated at 37°C for 30 min. After incubation, protein precipitation and redissolution procedures were identical to those described in the part of Section 2.2.2. Another 400 *μ*L of the same bile sample was processed with the same procedures only except without adding *β*-glucuronidase or sulfatase for incubation. 5 *μ*L of posttreated sample was injected in UPLC-Q/TOF MS for analysis. The effect of the glucuronidase or sulfatase was studied by comparing the UPLC-Q/TOF MS peak intensities for compounds of interests before and after the enzymatic incubation. The compounds of interest included glucuronidated conjugates or sulfated conjugates and their nonconjugated forms (hydrolyzed forms).

### 2.5. Chromatographic and Mass Spectrometry Conditions

 The chromatographic system used was Waters Acquity UPLC (Waters, Milford, MA, USA) equipped with binary solvent manager, sample manager, column manager, and PDA detector, which was also coupled with a Q/TOF mass spectrometer. For the separation of metabolites in biological samples, chromatographic analysis was performed with an acquity UPLC HSS C18 column (100 mm × 2.1 mm i.d., 1.8 *μ*m particle size, Waters Corporation, Milford, MA, USA). The column was eluted with gradient conditions: 0–10 min, linear from 98% to 92% A; 10–17 min, linear from 92% to 88% A; 17–22 min, linear from 88% to 5% A; 22–25 min, held at 5% A for 3 min; 25-26 min, linear from 5% to 98% A; 26–35 min, held at 98% A for 9 min to prepare equilibration of the column for next analysis, where mobile phase A consisted of 0.005% formic acid in de-ionized water and mobile phase B consisted of acetonitrile. The flow rate was 500 *μ*L/min and the column temperature was maintained at 40°C, while the sample-tray temperature was kept at 4°C.

A Waters acquity Synapt G2 quadrupole time-of-flight (Q/TOF) tandem mass spectrometry (Waters Corp., Manchester, UK) was connected to the UPLC system via an electrospray ionization (ESI) interface and controlled by MassLynx software (Version 4.1). The ESI source was operated in the negative ionization mode, and optimized conditions for maximum detection of metabolites were as follows: capillary voltage, 3.0 kV; sample cone, 25 V; extraction cone, 4 V; source temperature, 150°C; desolvation temperature, 450°C. The cone and desolvation gas (N_2_) flows were set at 50 and 850 (L/H). Leucine-enkephalin was used as the lock mass generating a reference ion in positive mode at *m/z* 556.2771 and introduced by a lockspray at 5 *μ*L/min for accurate mass acquisition.

The mass spectrometer and UPLC system were controlled by MassLynx 4.1 software. Data were collected in centroid mode, and the MS^E^ approach using dynamic ramp of collision energy was carried out in two scan functions—Function 1 (low energy): mass-scan range: 100–1000; scan time: 0.2 s; inter-scan delay: 0.05 s; collision energy: 4 V; Function 2 (high energy): mass-scan range: 100–1000; scan time: 0.2 s; inter-scan delay: 0.05 s; collision energy ramp of 15–30 V. MS/MS experiments were operated for major metabolites to obtain additional information from product ions. Comparison of fragment ion spectra between genipin and metabolites further aided in the identification of metabolite structures and site (s) of modifications in the parent molecule.

### 2.6. Isolation of the Major Metabolite from Rat Bile

Twenty male Sprague-Dawley rats weighing 180–220 g were given a single dose of genipin solution at 50 mg/kg of body weight by gavage into the stomach. After administration, the bile samples were collected by bile duct cannulation surgery. semipreparative HPLC (high performance liquid chromatography) was performed on an Agilent 1100 system consisting of a G1379A degasser, a G1311A quaternary pump, a 7725i manual sampler, and a G1316A thermostated column compartment with a G1315D DAD (Diode Array Detector) detector. Rat bile samples (100 mL) were first extracted three times by ethyl acetate and butyl alcohol. The ethyl acetate and butyl alcohol solution were removed later. The water soluble fraction was subjected to a macroporous absorption resin chromatography in an elution liquid (Ethanol/Water, 10/90 v/v). Metabolite G1 in elution was purified by semipreparative HPLC using an Agilent Eclipse XDB-C18 ODS column (250 mm × 9.4 mm, 5 *μ*m) and gradient conditions consisting of (A) acetonitrile and (B) water: 0–15 min, linear from 4% to 60% A at a flow rate of 3 mL/min to isolate the major metabolite in rat bile. Its purity would be determined by the high performance liquid chromatography-variable wavelength detector (HPLC-VWD) analysis. Its chemical structure was identified by comparison of their UV, IR, ESI-MS, ^1^H-NMR, and ^13^C-NMR spectra with the conference [13].

## 3. Results and Discussion

### 3.1. UPLC-Q/TOF MS Analysis of Genipin

**Figure 1:**
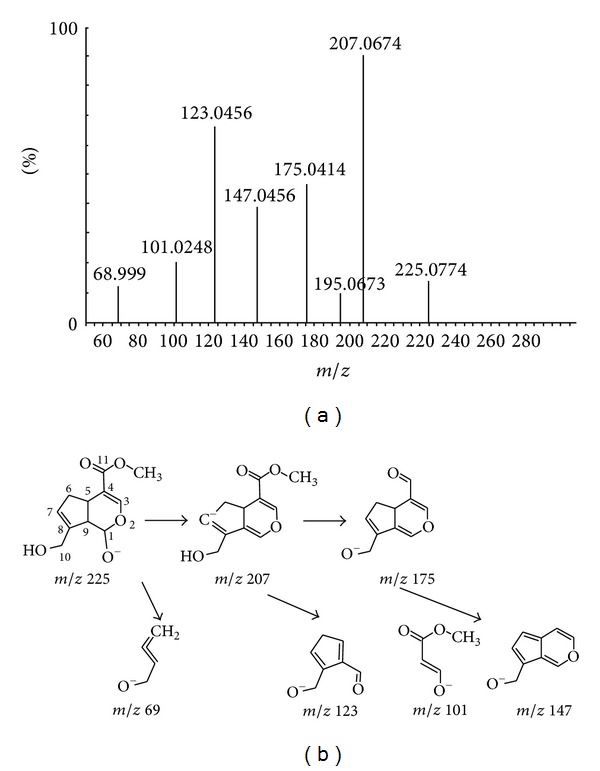
(a) Mass spectrum of genipin obtained on Q-TOF mass spectrometry at high collision energy. (b) Proposed fragmentation pathways of genipin.

 To identify the metabolites of genipin, the chromatographic and MS fragmentation behaviors of the parent compound genipin were first investigated. The retention time of genipin was 13.37 min under the chromatographic conditions employed. In the MS^2^ fragmentation mode, genipin formed a pseudomolecule weight of [M-H]^−^ at *m/z* 225.0774. The elemental compositions, double bond equivalents (DBEs), the experimental masses and calculated masses, and the mass errors of the pseudomolecular ion and its fragment ions were displayed in Table 1. The maximum of mass errors between measured and calculated values was less than 15 ppm (≤2.0 mDa), which signified high resolution and good accuracy.  Figure 1(a) showed the product ion spectrum of genipin under the high collision energy scan. On the basis of the high resolution mass spectral information, a tentative pathway for the formation of the most informative fragment ions of genipin is proposed in Figure 1(b). The product ion at *m/z* 207.0674 was generated by the loss of an OH radical (C-1) from the pseudomolecular ion at *m/z* 225.0774. The presence of a product ion at *m/z* 175.0414 was probably resulted from a cleavage of methylol group on C-11 from the ion at *m/z* 207.0674. And the major moiety ion at *m/z* 225.0774 may have undergone a successive loss of an OH radical on C-1 and a methyl ester on C-4 to produce the ion exhibiting *m/z* 147.0456. In addition, two other fragment ions gave *m/z* 123.0456 and *m/z* 101.0248 (Table 1), which could serve as characteristic ions for screening and identifying metabolites with similar skeletons. 

### 3.2. Identification of Metabolites in Rat Bile, Blood, Feces, and Urine

**Figure 2:**
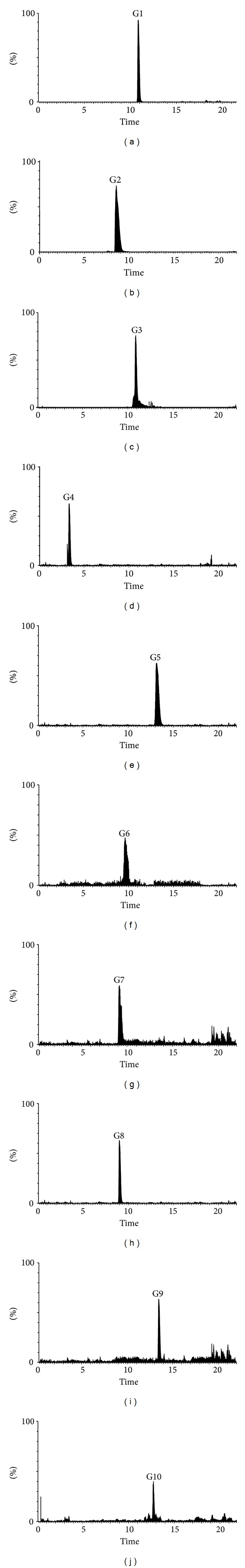
Extracted ion chromatograms of ten metabolites of genipin.

**Figure 3:**
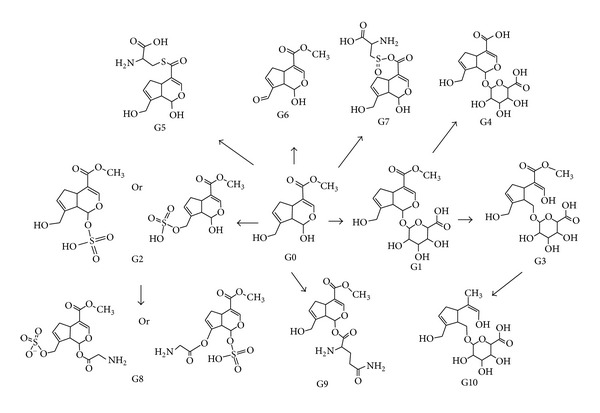
The possible metabolic pathways of genipin in rat bile.

The UPLC and MS conditions were optimized to obtain a full overview of metabolites by comparing the drug-containing biological samples with blank biological samples. As shown in Figure 2, ten metabolites of genipin were detected in rat bile samples. Extracted ion chromatogram of genipin and its metabolites are presented in Figure 3.  Table 2 lists the detailed information of these metabolites, including the retention times, proposed elemental compositions, and the characteristic fragment ions.

*Metabolite G1*. G1 was the most abundant metabolite in rat's bile on the basis of the chromatographic peak area. It was eluted at 10.95 min with a molecular weight [H-1]^−^ of 401.1102 and a derived formula of C_17_H_21_O_11_. High collision energy analysis revealed a characteristic product ion at 225.0765 with neural loss of a glucuronide unit with 176 Da in the MS/MS spectra. Fragment ions at *m/z* 207.0673, 147.0450, 123.0450, and 101.0247 were the same as those of genipin. Therefore, G1 was identified as monoglucuronidated conjugate of genipin. But the accurate site of glucuronated action could not be confirmed because it could be occurred on the hydroxyl groups of both C-1 and C-10 positions. 

*Metabolite G2*. G2 had a retention time of 8.76 min and showed an [M-H]^−^ ion at *m/z* 305.0348, corresponding to the elemental composition of C_11_H_13_SO_8_. In the MS/MS spectra of G2, the fragment ion at *m/z* 225.0767 was generated from the [M-H]^−^ ion by the loss of a sulfate unit with 80 Da from metabolite G2. And high collision energy analysis revealed product ions at 207.0668, 147.0445, 123.0461, and 101.0254 that were the same as those of genipin. Metabolite G2 was tentatively identified as monosulfated conjugate of genipin. 

*Metabolite G3*. G3 was eluted at 9.74 min and displayed a molecular ion at *m/z* 403.1197. Accurate mass measurement showed that the chemical formula was C_17_H_23_O_11_, suggesting the addition of two hydrogen atoms on metabolite G1. The product ion at *m/z* 227.0595 was the base peak in the MS/MS spectrum, with a loss of 176 Da, indicating that a glucuronic acid was attached to the aglycone moiety. The product ions of G3 at *m/z* 227.0595, 209.0769, and 149.0576 were all 2 Da greater compared with product ions *m/z* 225.0767, 207.0668, and 147.0445 from genipin. Hence, it could be inferred that metabolite G3 was ring-opened derivative of G1. The glucuronated action can be occurred on the hydroxyl groups of both C-1 and C-10 positions. However, the site of the glucuronated action could not be characterized from the MS/MS spectra. 

*Metabolite G4*. G4 was eluted at 3.55 min and displayed a molecular ion at *m/z* 387.0903. Accurate mass measurement showed that the chemical formula was C_16_H_19_O_11_, suggesting the loss of CH_2_ from metabolite G1. The product ions of G4 at *m/z* 211.0586 and 193.0312 were all 14 Da lower compared with product ions of genipin at *m/z* 225.0767 and 207.0668. Moreover, the character product ion of genipin at *m/z* 147.0461 was also observed in the MS/MS spectrum of metabolite G4. Hence, it could be inferred that metabolite G4 was demethylation derivative of metabolite G1. 

**Figure 4:**
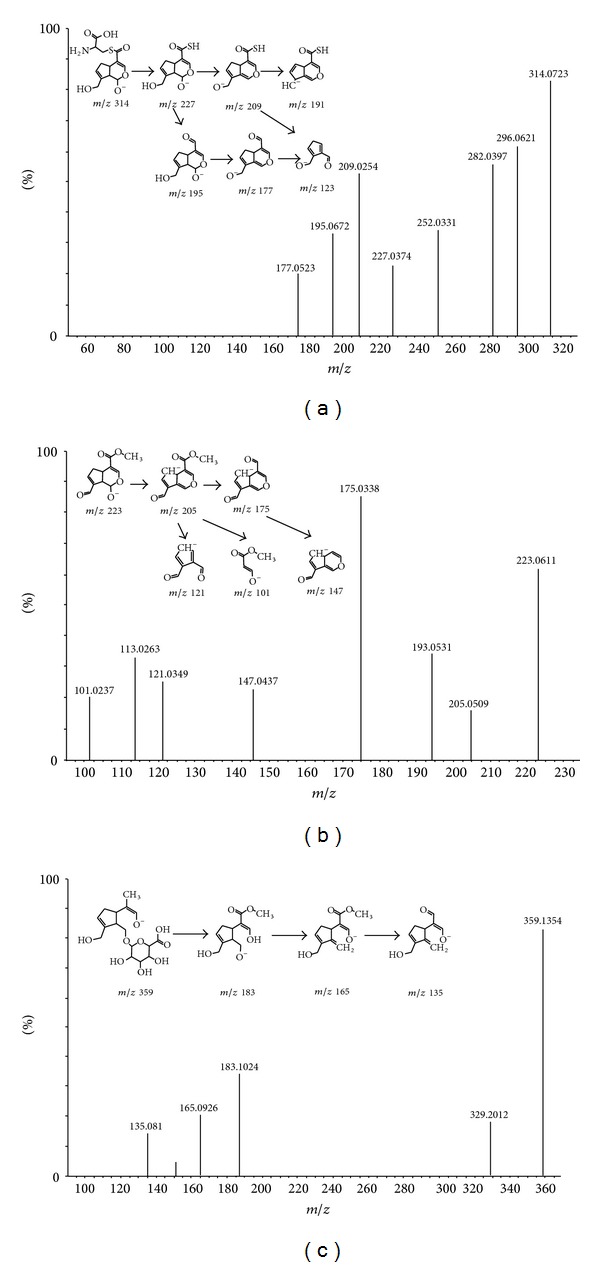
Representative MS/MS spectrum of [M-H]^−^ ion at *m/z* 314.0723 for metabolite G5 (a); MS/MS spectrum of [M-H]^−^ ion at *m/z* 223.0611 for metabolite G6 (b); MS/MS spectrum of [M-H]^−^ ion at *m/z* 359.1354 for metabolite G10 (c).

*Metabolite G5*. G5 with a retention time at 13.01 min had a molecular ion ([M-H]^−^) at *m/z* 314.0723. Accurate mass measurement showed that the chemical formula of G5 was C_13_H_16_NO_6_S. The molecular ion at *m/z* 314.0723 could have lost an H_2_O molecule to generate a product ion at *m/z* 296.0621. It was notable that [M-H]^−^ ion of G5 could generate a product ion at *m/z* 195.0672 by losing a C_3_H_5_NO_2_S unit. It indicated that there was a cysteine group in G5. High collision energy analysis revealed product ions at 227.0374, 209.0254, 195.0672, and 177.0523 with the proposed fragmentation pathways of G5 was shown in Figure 4(a). Hence, G5 was identified as cysteine conjugate of demethylol-genipin. 

*Metabolite G6*. G6 had a retention time of 9.60 min and showed an [M-H]^−^ ion at *m/z* 223.0611, corresponding to the elemental composition of C_11_H_11_O_5_. As shown in Table 3, the molecular ion of G6 lost H_2_O, a molecule of methoxy, and aldehyde to form product ions at *m/z* 205.0509, *m/z* 193.0531, and 147.0437. The proposed fragmentation pathways of G6 were shown in Figure 4(b). Hence, G6 was identified as 10-aldehyde-genipin. 

*Metabolite G7*. G7 had a retention time of 9.26 min and showed an [M-H]^−^ ion at *m/z* 346.0406, corresponding to the elemental composition of C_13_H_16_NO_8_S. The [M-H]^−^ ion of G7 could lose H_2_O to generate the fragment ion at *m/z* 328.0516 and a molecule of HCOOH subsequently to generate the fragment ions at *m/z* 284.0632. And another product ion at *m/z* 211.0614 was generated by losing C_3_H_5_NO_3_S from the precursor ion at *m/z* 346.0406. Fragment ions at *m/z* 175.0411 and 123.0444 were the same to those from genipin. Hence, G7 was identified as cysteinesulfinic acid conjugate of demethylol-genipin. 

*Metabolite G8*. G8 had a retention time of 9.21 min and showed an [M-H]^−^ ion at *m/z* 362.0593, corresponding to the elemental composition of C_13_H_16_NO_9_S. In the MS/MS spectra of G8, the fragment ion at *m/z* 305.0350 was generated from the [M-H]^−^ ion by the loss of C_2_H_3_NO unit from Metabolites G8. The major fragment ion at *m/z* 305.0350 was the same as that of G2. Moreover, fragment ions at *m/z* 207.0655, 123.0447, and 101.0241 were same to those from G2. Therefore, G8 was identified as glycine conjugate of G2. 

*Metabolite G9*. G9 had a retention time of 13.59 min and showed an [M-H]^−^ ion at *m/z* 353.1381, corresponding to the elemental composition of C_16_H_21_N_2_O_7_. The [M-H]^−^ ion of G9 could lose H_2_O to generate the fragment ion at *m/z* 335.1288. Moreover, the character product ions of genipin at *m/z* 207.0674, 175.0414, and 147.0456 were also observed in the MS/MS spectrum of metabolite G9. Hence, it could be inferred that metabolite G9 was glutamine conjugate of genipin. 

*Metabolite G10*. A minor compound with the retention time of 12.7 min in the chromatogram was observed as metabolite G10 with a molecule ion at *m/z* 359.1354, corresponding to the elemental composition of C_16_H_23_O_9_. The product ion at *m/z* 183.1024 was also observed in the MS/MS spectrum, with a loss of 176 Da, indicating that a glucuronic acid was attached to the aglycone moiety. The product ion at *m/z* 165.0926 of G10 was formed by the loss of an H_2_O unit from the product ion *m/z* 183.1024, followed by the loss of one CH_2_O unit to form *m/z* 135.0810. The proposed fragmentation pathways of G10 were shown in Figure 4(c).

After analysis of the metabolites of genipin in rats urine, feces, and plasma samples, the result showed that all metabolites excluding G1 to G10 appeared in other samples could be detected in bile. The result was shown in Table 3. There were six metabolites (G1, G2, G3, G5, G7, and G9) in urine, two metabolites (G1 and G2) in plasma, and one metabolite (G1) in feces. And the plasma samples from rats which were administrated genipin by intravenous administration in the fourth group were also analyzed. Only one metabolite (G1) was identified whereas the parent form of genipin was absent. 

In this metabolism study of genipin, it could conclude that genipin was an active compound that could be metabolized to other forms wholly and immediately. On the basis of the chromatographic peak area, the sulfated and glucuronidated conjugates of genipin were major metabolites and there were other 8 metabolites. According to the result, demethylated, ring-opened, cysteine-conjugated, hydroformylated, glucuronidated, and sulfated transformations were proposed to be the possible metabolic pathways of genipin in rat, which would improve our knowledge about the *in vivo* metabolism of genipin.

### 3.3. Metabolism of Genipin *In Vitro* in Human Liver Microsomes

Incubations of genipin in human liver microsomes (HLM) protein were performed to determine the hepatic contribution to the overall disposition of this drug. Comparing with the three negative control samples, metabolite G1 was detected in HLM incubations with the amount of genipin decreasing. Using the UPLC-QTOF/MS, metabolite G1 had a retention time of 10.95 min and showed an [M-H]^−^ ion at *m/z* 401.1102 with fragment ions at *m/z* 207.0673, 147.0450, 123.0450, and 101.0247. Glucuronidation represents one of the major pathways for drug metabolism in humans and other mammalian species. Here, we found that genipin was metabolized in human liver microsomes immediately as the metabolite G1 that could be detected after 1 h incubation. And amount of metabolite G1 could not increase indicated that incubations of genipin in human liver microsomes (HLM) protein had been finished in 6 h. The result also confirmed that glucuronidated transformations were proposed to be the possible major metabolic pathways of genipin in rats.

### 3.4. Enzymatic Hydrolysis Analysis

**Figure 5:**
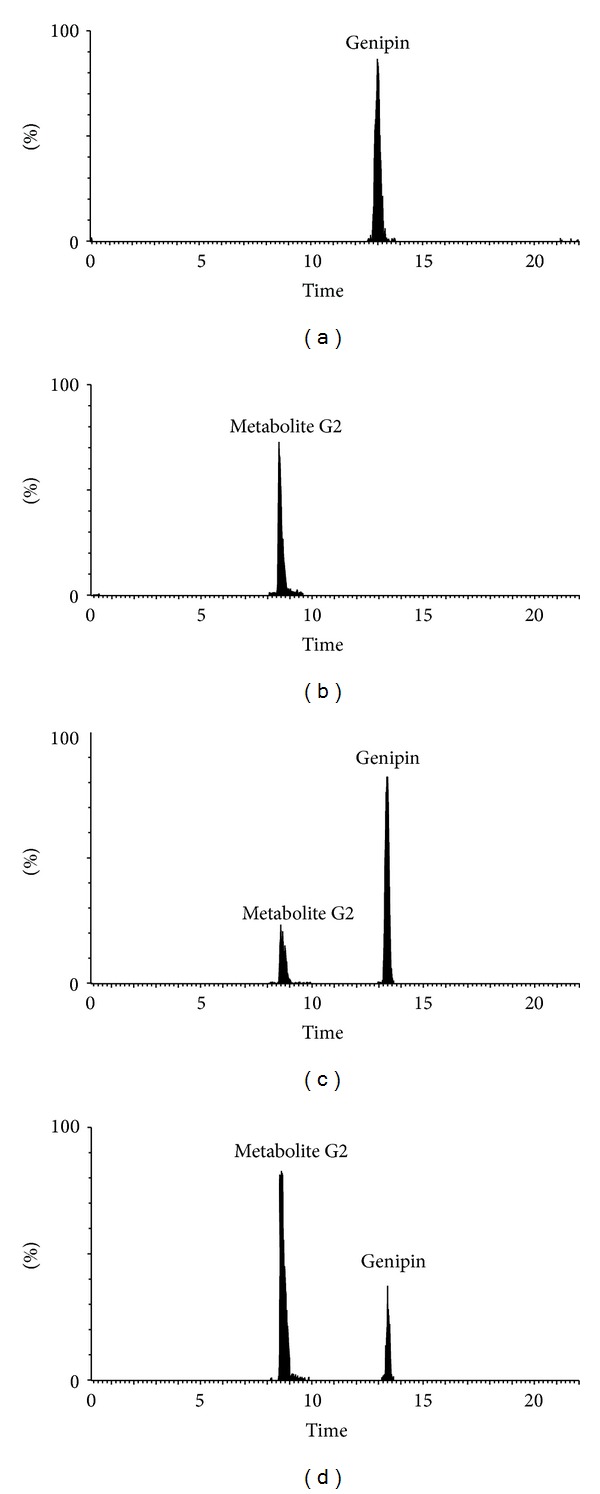
The extracted ion chromatogram of *m/z *225 of bile treated accordingly to experimental schemes. (a) the standard solution with genipin at concentration of 50 ng/mL; (b) the drug-containing bile; (c) the drug-containing urine hydrolyzed by sulfatase; (d) the drug-containing urine hydrolyzed with *β*-glucuronidase.

**Figure 6:**
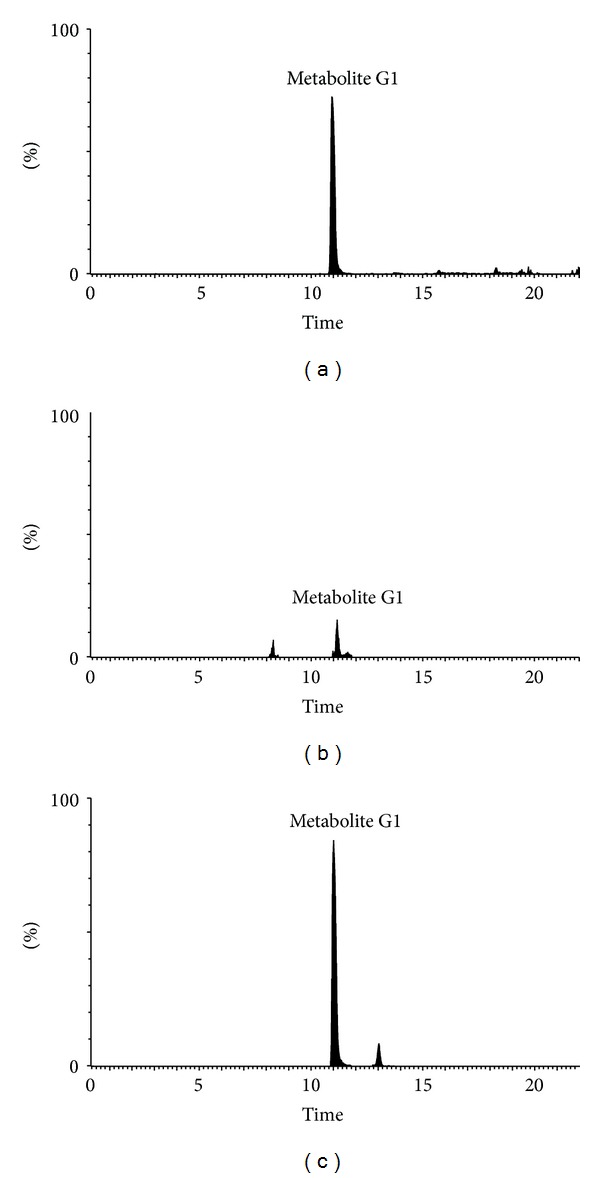
The extracted ion chromatogram of *m/z *401 of bile treated accordingly to experimental schemes. (a) the drug-containing bile; (b) The drug-containing urine hydrolyzed by *β*-glucuronidase; (c) the drug-containing urine hydrolyzed with sulfatase.

**Figure 7:**
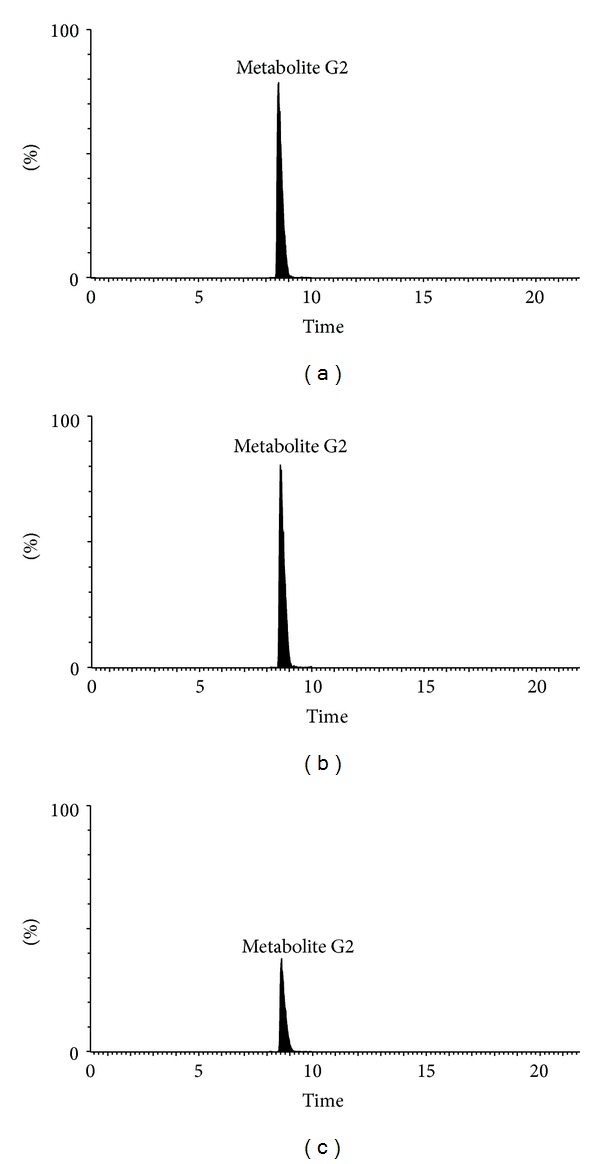
The extracted ion chromatogram of *m/z *305 of bile treated accordingly to experimental schemes. (a) the drug-containing bile; (b) The drug-containing urine hydrolyzed by *β*-glucuronidase; (c) the drug-containing urine hydrolyzed with sulfatase.

The metabolism study of genipin showed that the parent form of genipin could not be detected in biological samples (bile, urine, plasma, and feces). In order to confirm the existence of major metabolite G1 and G2, the drug-containing urine samples were hydrolyzed with *β*-glucuronidase or sulfatase. After UPLC-QTOF/MS analysis, the appearance of genipin (*m/z* 225.0774) with the retention time at 13.25 min (Figure 5) was all detected in the drug-containing urine samples after *β*-glucuronidase or sulfatase hydrolysis. When *β*-glucuronidase was added to urine samples with the same processing, metabolite G1 was hydrolyzed to genipin as metabolite G2 could not be hydrolyzed by *β*-glucuronidase (Figure 6). On the contrary, when sulfatase was added to urine samples with the same processing, some amount of metabolite G2 could be hydrolyzed to genipin which was showed with the decrease of the peak area of metabolite G2 (Figure 7). 

### 3.5. Identification of the Structure of Metabolite G1

**Figure 8:**
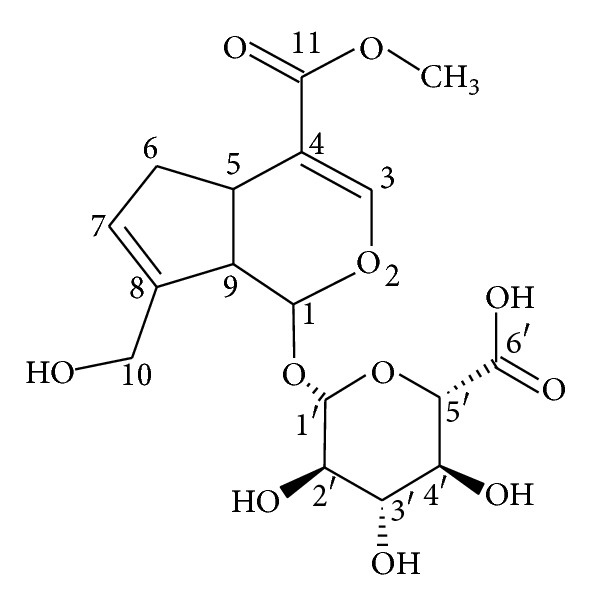
Chemical structure of genipin-1-o-glucuronic acid.

**Figure 9:**
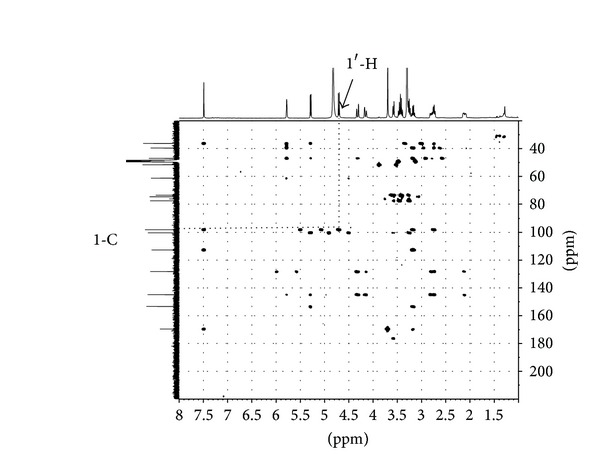
Key HMBC correlations of genipin-1-o-glucuronic acid. The coupling from 1′-H to 1-C was observed, which confirms the attachment of a glucuronic acid group to 1-C.

Metabolite G1 was the most abundant metabolite in rat bile on the basis of the chromatographic peak area, and it was isolated from rats bile as described in Section 2.6. From the following experimental data, it was confirmed that metabolite G1 was genipin-1-o-glucuronic acid. Its structure was shown in Figure 8. IR *v*_max⁡_^KBr^ cm^−1^: 3400, 1710, 1630. UV *λ*_max⁡_^MeOH^ (log⁡*ε*): 240 nm (4.96). ^1^H NMR (400 MHz, CD3OD): *δ*7.49 (s, 1H, 3-H), 5.78 (s, 1H, 7-H), 5.28 (d, *J* = 7.2 Hz, 1H, 1-H), 4.70 (d, *J* = 7.9, 1H, 1′-H), 4.32 (d, *J* = 14.4 Hz, 1H, 10-Ha), 4.15 (d, *J* = 14.8 Hz, 1H, 10-Hb), 3.70 (s, 3H, –OCH_3_), 3.58 (d, *J* = 9.2 Hz, 1H, 5′-H), 3.48–3.39 (m, 2H, 3′-H, 4′-H), 3.27–3.14 (m, 2H, 2′-H, 5-H), 2.82–2.72 (m, 2H, 6-Ha, 9-H), 2.14–2.08 (m, 1H, 6-Hb); ^13^C NMR (100 MHz, CD3OD): *δ*36.35 (5-C), 39.64 (6-C), 47.06 (9-C), 51.68 (OCH3), 61.32 (10-C), 73.56, 74.71, 76.26, 77.62 (2′~5′-C), 98.07 (1-C), 100.32 (1′-C), 112.65 (4-C), 128.23 (7-C), 144.86 (8-C), 153.36 (3-C), 169.59 (11-C), 176.5 (6′-C). The HMBC correlation of genipin-1-o-glucuronic acid was shown in Figure 9.

## 4. Conclusions

In the present study, metabolism of genipin in the rat was extensively studied by using UPLC-QTOF/MS. Accurate masses along with mass fragmentation were applied to elucidate the structures of metabolites with the aid of MetaboLynx software tools. Ten metabolites (G1–G10) were found in rat bile, and their structures were elucidated based on the retention times on the UPLC system, the accurate molecular mass, and characteristic fragment ions. And six metabolites (G1, G2, G3, G5, G7, and G9) in urine, two metabolites (G1 and G2) in plasma, and one metabolite (G1) in feces were detected. Among the ten metabolites, the sulfated and glucuronidated conjugates of genipin were major metabolites on the basis of the chromatographic peak area. Demethylated, ring-opened, cysteine-conjugated, hydroformylated, glucuronidated, and sulfated transformations were proposed to be the possible metabolic pathways of genipin in rat.

In the *in vitro* experiment, genipin can be transformed to metabolite G1 after incubation in human liver microsomes with UDP-glucuronosyltransferases. In the drug-containing bile samples, metabolite G1 could be hydrolyzed to genipin by *β*-glucuronidase, and metabolite G2 could be hydrolyzed to genipin by sulfatase. It confirmed the existence of major metabolite G1 and G2 in the drug-containing bile samples. 

At last, the major metabolite G1 was isolated by a semipreparative HPLC using an Agilent Eclipse XDB-C18 ODS column. Its purity was up to 98% which was determined by the high performance liquid chromatography-variable wavelength detector (HPLC-VWD) analysis. And its chemical structure was identified by comparison of their UV, IR, ESI-MS, ^1^H-NMR, and ^13^C-NMR spectra with the conference. Metabolite G1 was confirmed as genipin-1-o-glucuronic acid.

## Figures and Tables

**Table 1:** The predicted elemental compositions, measured masses and calculated masses, double-bond equivalents (DBEs), and mass errors of pseudo molecule of genipin and its fragment ions.

Elemental composition	Measured mass (Da)	Calculated mass (Da)	DBE	Error (mDa)	Error (ppm)
C_11_H_13_O_5_^−^	225.0774	225.0763	4.5	1.1	4.9
C_11_H_11_O_4_^−^	207.0674	207.0657	6.5	1.7	8.2
C_10_H_11_O_4_^−^	195.0673	195.0657	5.5	1.6	8.2
C_10_H_7_O_3_^−^	175.0414	175.0395	7.5	1.9	10.9
C_9_H_7_O_2_^−^	147.0456	147.0446	6.5	1.0	6.8
C_7_H_7_O_2_^−^	123.0456	123.0446	4.5	1.0	8.1
C_4_H_5_O_3_^−^	101.0248	101.0239	2.5	0.9	8.9

**Table 2:** The retention times, accurate measurements, and elemental formula of protonated molecules and product ions by Q-TOF MS/MS analysis of metabolites.

Compound no.	*t*_*R*_ (min)	Molecular ions *m*/*z* (Da)	Fragment ions *m*/*z* (Da)	Formula	Error (mDa)	Error (ppm)
G0 (genipin)	13.37	225.0774		C_11_H_13_O_5_	1.1 mDa	4.9 ppm
			207.0674	C_11_H_11_O_4_	1.7 mDa	8.2 ppm
			195.0673	C_10_H_11_O_4_	1.6 mDa	8.2 ppm
			175.0414	C_10_H_7_O_3_	−1.5 mDa	−8.6 ppm
			147.0456	C_9_H_7_O_2_	1.0 mDa	6.8 ppm
			123.0456	C_7_H_7_O_2_	1.3 mDa	10.6 ppm
			101.0248	C_4_H_5_O_3_	0.9 mDa	8.9 ppm

G1	10.95	401.1102		C_17_H_21_O_11_	1.8 mDa	4.5 ppm
			383.1001	C_17_H_19_O_10_	2.3 mDa	6.0 ppm
			369.0831	C_16_H_17_O_10_	0.9 mDa	2.4 ppm
			225.0765	C_11_H_13_O_5_	0.2 mDa	0.9 ppm
			207.0673	C_11_H_11_O_4_	1.6 mDa	7.7 ppm
			147.0450	C_9_H_7_O_2_	0.4 mDa	2.7 ppm
			123.0450	C_7_H_7_O_2_	0.3 mDa	3.3 ppm
			101.0247	C_4_H_5_O_3_	0.8 mDa	7.9 ppm

G2	8.76	305.0348		C_11_H_13_SO_8_	1.7 mDa	5.6 ppm
			287.0241	C_11_H_11_SO_7_	1.6 mDa	5.6 ppm
			273.0085	C_10_H_9_SO_7_	1.6 mDa	5.9 ppm
			225.0767	C_11_H_13_O_5_	0.4 mDa	1.8 ppm
			207.0668	C_11_H_11_O_4_	1.1 mDa	5.3 ppm
			147.0445	C_9_H_7_O_2_	−0.1 mDa	−0.7 ppm
			123.0461	C_7_H_7_O_2_	1.0 mDa	8.1 ppm
			101.0254	C_4_H_5_O_3_	0.5 mDa	4.9 ppm

G3	9.74	403.1197		C_17_H_23_O_11_	4.3 mDa	−10.7 ppm
			227.0595	C_11_H_15_O_5_	−3.1 mDa	13.7 ppm
			209.0769	C_11_H_13_O_4_	−4.5 mDa	9.1 ppm
			197.0808	C_10_H_13_O_4_	−0.6 mDa	−3.0 ppm
			177.0552	C_10_H_9_O_3_	0.1 mDa	0.6 ppm
			149.0576	C_9_H_9_O_2_	−2.7 mDa	−18.1 ppm

G4	3.55	387.0903		C_16_H_19_O_11_	−2.4 mDa	−6.2 ppm
			343.1046	C_15_H_19_O_9_	1.7 mDa	5.0 ppm
			325.0938	C_15_H_17_O_8_	1.5 mDa	4.6 ppm
			211.0586	C_10_H_11_O_5_	−2.0 mDa	−9.5 ppm
			193.0312	C_10_H_9_O_4_	−1.1 mDa	−5.7 ppm
			175.0404	C_10_H_7_O_3_	−0.9 mDa	−5.1 ppm
			147.0461	C_9_H_7_O_2_	1.5 mDa	10.2 ppm

G5	13.01	314.0723		C_13_H_16_NO_6_S	2.5 mDa	8.0 ppm
			296.0621	C_13_H_14_NO_5_S	2.8 mDa	9.5 ppm
			282.0397	C_12_H_12_NO_5_S	−3.9 mDa	−13.8 ppm
			264.0335	C_12_H_10_NO_4_S	0.4 mDa	1.5 ppm
			252.0331	C_11_H_10_NO_4_S	2.0 mDa	7.9 ppm
			227.0374	C_10_H_11_O_4_S	−0.4 mDa	−1.8 ppm
			209.0254	C_10_H_9_O_3_S	−1.8 mDa	−8.6 ppm
			195.0672	C_10_H_11_O_4_	1.5 mDa	7.7 ppm
			177.0523	C_10_H_9_O_3_	−0.9 mDa	−5.1 ppm

G6	9.60	223.0611		C_11_H_11_O_5_	0.5 mDa	2.2 ppm
			205.0509	C_11_H_9_O_4_	0.8 mDa	3.9 ppm
			193.0531	C_10_H_9_O_4_	0.8 mDa	3.9 ppm
			175.0388	C_10_H_5_O_3_	−0.7 mDa	−4.0 ppm
			147.0437	C_9_H_7_O_2_	−0.9 mDa	−6.1 ppm
			121.0299	C_7_H_5_O_2_	0.9 mDa	7.4 ppm
			101.0237	C_4_H_5_O_3_	−0.2 mDa	−2.0 ppm

G7	9.26	346.0406		C_13_H_16_NO_8_S	−0.1 mDa	−0.3 ppm
			328.0516	C_13_H_14_NO_7_S	2.5 mDa	7.6 ppm
			284.0632	C_12_H_14_NO_5_S	2.5 mDa	7.6 ppm
			252.0351	C_11_H_10_NO_4_S	2.0 mDa	7.9 ppm
			211.0614	C_10_H_11_O_5_	0.8 mDa	3.8 ppm
			193.0504	C_10_H_9_O_4_	0.3 mDa	1.6 ppm
			175.0411	C_10_H_7_O_3_	1.6 mDa	9.1 ppm
			123.0444	C_7_H_7_O_2_	−0.2 mDa	−1.6 ppm

G8	9.21	362.0593		C_13_H_16_NO_9_S	4.3 mDa	13.0 ppm
			305.0350	C_11_H_13_SO_8_	1.9 mDa	6.2 ppm
			287.0219	C_11_H_11_SO_7_	−0.6 mDa	−2.1 ppm
			207.0655	C_11_H_11_O_4_	−0.2 mDa	−1.0 ppm
			123.0447	C_7_H_7_O_2_	0.1 mDa	0.8 ppm
			101.0241	C_4_H_5_O_3_	0.2 mDa	2.0 ppm

G9	13.59	353.1381		C_16_H_21_N_2_O_7_	3.2 mDa	9.1 ppm
			335.1288	C_16_H_19_N_2_O_6_	4.5 mDa	13.4 ppm
			207.0674	C_11_H_11_O_4_	1.7 mDa	8.2 ppm
			195.0673	C_10_H_11_O_4_	1.6 mDa	8.2 ppm
			175.0414	C_10_H_7_O_3_	−1.5 mDa	−8.6 ppm
			147.0456	C_9_H_7_O_2_	1.0 mDa	6.8 ppm

G10	12.7	359.1354		C_16_H_23_O_9_	1.2 mDa	3.3 ppm
			183.1024	C_10_H_15_O_3_	0.3 mDa	1.6 ppm
			165.0926	C_10_H_13_O_2_	1.0 mDa	6.1 ppm
			135.0810	C_9_H_11_O	0.9 mDa	6.7 ppm

**Table 3:** The metabolites in different biological samples of rats.

	Formula	Bile	Urine	Plasma*	Feces	Plasma**
G0	C_11_H_14_O_5_ (genipin)	−	−	−	−	−
G1	C_17_H_22_O_11_	+	+	+	−	+
G2	C_11_H_14_SO_8_	+	+	+	+	−
G3	C_17_H_24_O_11_	+	+	−	−	−
G4	C_16_H_20_O_11_	+	−	−	−	−
G5	C_13_H_17_NO_6_S	+	+	−	−	−
G6	C_11_H_12_O_5_	+	−	−	−	−
G7	C_13_H_17_NO_8_S	+	+	−	−	−
G8	C_13_H_17_NO_9_S	+	−	−	−	−
G9	C_16_H_22_N_2_O_7_	+	+	−	−	−
G10	C_16_H_24_O_9_	+	−	−	−	−

+: found; −: not found.

Plasma* were from rats given single dose of genipin solution at 50 mg/kg of body weight by gavage into the stomach.

Plasma** were from rats given single dose of genipin solution at 10 mg/kg of body weight by intravenous administration.
